# The role of Islamic Ruqyah at end-of-life: An opportunity to provide metaphysical relief

**DOI:** 10.1017/S1478951525000434

**Published:** 2025-05-23

**Authors:** Megan Thorvilson, Faduma Warsame, Asmaa Ferdjallah

**Affiliations:** 1 Pediatric Palliative Medicine, Mayo Clinic, Rochester, MN, USA; 2 Department of Spiritual Care, Mayo Clinic, Rochester, MN, USA; 3 Pediatric Hematology and Oncology, Mayo Clinic, Rochester, MN, USA

**Keywords:** Ruqyah, end-of-life, Islam, Western medicine, culture

## Abstract

**Objective:**

Practitioners in the West care for patients from diverse backgrounds. For Muslim patients who experience end-of-life in a foreign society, it is especially prudent to provide access to cultural and religiously appropriate practices. The Quran, the Islamic Holy book, is a key central aspect in the life of a Muslim. Ruqyah, that is – recitation of the Quran, is an often unrealized and misunderstood facet to a peaceful end-of-life for Muslim patients receiving palliative care. Ruqyah may offer comfort and be a source of relief for some Muslims but may be misunderstood as a lack of acceptance of impending death.

**Methods:**

This case report and single patient chart review describes the use of Ruqyah at the end-of-life and the role of Western practitioners as it relates to this practice. A critical analysis was undertaken to address the themes of hope, spirituality, and autonomy at end-of-life followed by a literature review.

**Results:**

Maintaining a sense of hope is a religious duty albeit one in which the outcome is not within the believer’s hands. For Muslims, to hope is to believe – which is to accept death when it arrives. In a varied world with rich cultures, it is fundamental for end-of-life providers to incorporate cultural or religious rituals into their working knowledge of the dying process.

**Significance of results:**

This case demonstrates the importance of the basic understanding of Islamic end-of-life practices in conjunction with Muslim spiritual and chaplaincy resources.

## Introduction

Death and dying are deeply personal experiences often shaped by culture and religion. For Muslims who receive intensive medical care in the West, there may be a disconnect between Islamic principles of death and Western goals of care (Tayeb et al. 2010). Though well meaning, Western attitudes may be in direct conflict with establishing a harmonious death (Al-Shahri and Al-Khenaizan 2005). Here, we present the case of a Muslim young woman from Saudi Arabia who traveled to the United States for advanced therapy and ultimately died. Her end-of-life course was characterized by provider angst and inability to provide a key ritual known as Ruqyah as a part of palliative care. This case highlights the need to ensure patient-centered care and an accommodation for end-of-life practices for Muslim patients.

## Case

A 16-year-old girl from Saudi Arabia with refractory acute myeloid leukemia despite multiple lines of therapy, traveled with her family to the United States to pursue continued therapy. The family, who was of Muslim Sunni background, hoped to pursue cure with a hematopoietic stem cell transplant (HSCT). Despite multiple cycles of novel and directed chemotherapy agents to induce a remission to be eligible for HSCT, she died after 5 months of therapy without undergoing HSCT. Due to the aggressive nature of her underlying disease and poor prognostic outcome, both Palliative Care and Chaplaincy services were involved early and throughout her course as a matter of institutional routine.

In the months preceding her death, the family and patient clearly asserted their religious beliefs and trust in God. As her disease progressed, the patient eloquently advocated for her wishes regarding care at end-of-life, sharing her hope to avoid sedation and intubation. The family worked diligently to minimize her pain and discomfort while balancing their hope for awake and alert times. The family requested discussions of critical medical decisions away from the patient to preserve her dignity. Although not typically pursued at the outset of care, a consultation with Ethics was pursued due to the provider team’s desire to confirm that the patient’s wishes continued to be heard. The patient was found to have the intellectual ability and maturity to share her voice and ultimately elected to defer her care decisions to her parents. She expressed a strong belief in Islam with a desire to continue to practice the tenets of her faith throughout her treatment and end-of-life care and ultimately delegated the details of practice to her parents.The patient’s father, who emerged as the primary decision-maker, struggled to balance his cultural and religious values of not removing or declining therapies that may prolong life while not prolonging his daughter’s suffering.

When it became clear that the patient’s disease was recurrent and incurable, the father clarified that the goals of care were ongoing intensive interventions with the goal to transition to comfort care only when she was in the active dying process. The mother shared that her home Muslim community was praying for the family and patient and performing charitable acts in honor of them. A request for Islamic Ruqyah was made to the chaplaincy service which proved difficult despite multidisciplinary efforts from chaplaincy, psychology, ethics, and the palliative care team. After much effort from the patient’s father, Ruqyah was performed during which the patient demonstrated both agitation and emesis, which were interpreted to reflect spiritual affliction and/or evil eye.

As it became clear that the patient was nearing the end of her life, her code status was revised to DNR/DNI to honor her wishes. An attempt was made to allow the family to travel with the patient back to their native Saudi Arabia, but she was deemed too medically unstable to travel. Ultimately, the patient was allowed a natural death in the hospital in the United States.

## Discussion

This case describes the death of a patient with aggressive leukemia who was from a deeply religious Muslim family. Although the family sought care in the West to receive the highest level of medical care, they did not have adequate access to important religious and cultural services while in the United States. For example, the role of Ruqyah was vastly misunderstood and the medical team was not able to assist in arranging this important piece of palliative care. Furthermore, an Islamic-based approach to death and dying was at times uncomfortable for Western health-care workers.

Islamic principles make it clear that death is a way of life, and that God is ultimately and fully in control of when human life comes to an end. Al-Qadr, or predestination, is a major tenet of Islam and is guided by evidence in the Quran, the Islamic Holy book, where it is stated “The Lord has created and balanced all things and has fixed their destinies and guided them” (The Holy Quran). For religious Muslims, this trust in God highlights a deeper hope – one that is ever present and always informed by God’s presence (Ibrahim 2003). While Muslim patients, like many patients, may opt for all reasonable medical interventions to prolong life – they are also painfully aware of the possible futility of these actions. Even so, the concept of palliative care and end-of-life can take a precarious role at the end of a Muslim’s life (Oakley 2023; Suleman 2023). Emphatically, Islam forbids any act that may hasten death – a concept that unfortunately forms the basis of the double effect (Browne et al. 2021; Oakley 2023). This fierce avoidance of interference with the timeline of end-of-life care may create a tension between achieving comfort and allowing the natural progression of death.

During the end of this patient’s life, there was a strong desire by the family to perform an Islamic ritual known as Ruqyah. Ruqyah is the act of providing Quranic recitation and prayer in the presence of an ill individual to support healing (Mayberry 2022). It is well described and oft prescribed in the Quran as in Chapter 17 – “Al-Isra” or “The Night Journey” – “And We send down in the Quran that which is a cure and a mercy for the believers” (The Holy Quran). The Prophet Muhammed, a central and important figure in Islam, was quoted as saying, “Make good use of the two cures; honey and the Quran” (Ibn Majah Al-rabi Al-qazwin and Al-Khattab 2007). He was known to utilize Ruqyah during his lifetime during periods of illness (Muslim and Al-Khattab 2007). The Quran and prayers can be recited by an Imam, family, community members, or even by an audio recording of a skilled Quranic reciter (Mayberry 2022). The act of listening to Quranic recitation and associated prayers that seek refuge in and call out to God as an invocation for healing is paramount to the ritual. Ruqyah is believed to reduce the incidence of mental anguish or distress, heal a physical ailment, and esoterically remove possession by evil eye and black magic.

For this patient, the intended request held even deeper importance – Ruqyah to provide a cure for her physical ailments. In Saudi Arabia, the practice of Ruqyah is well regulated and the government requires eligible practitioners to apply for a license. The Ministry’s Religious Guidance and Awareness Department approves each request for Ruqyah and a member of the Religious Guidance Team accompanies and supervises the Ruqyah healer. The healer is forbidden from prescribing medications or intervening in the medical treatment plan (Health Ministry to certify spiritual healers 2016). As Ruqyah is firmly rooted in Islamic tradition, although performed widely across the Islamic World, there is no variation in practice (Elkadi 1985). Specific cultures inside Muslim countries may disagree as to the importance or role of Ruqyah, but the practice itself is standardized (Salim 2004).

Although Ruqyah occurred during a period of declining status due to bacteremia, neutropenic colitis, increasing respiratory distress, and ICU delirium, for the family, the physical manifestations of agitation and emesis may have represented a spiritual affliction for which the remedy would be continued Ruqyah. As Ruqyah is often performed at or near the time of death, it is hard to state with certainty what the witnessed abnormality was attributed to. More importantly, the cause may be insignificant in a patient receiving end-of-life palliative care. The duty to maleficence takes a different form through the lens of each individual involved in this patient’s care. For the medical team, the news of her physical agitation and emesis may have been distressing if believed to be caused by Ruqyah, while for the family, this news might have brought relief. Although counterintuitive, those practicing Ruqyah are meant to continue recitation especially in the face of restlessness, agitation, and/or emesis. This highlights the diverging perspectives of secular and religious healing.

Despite its meaningful role at end-of-life, the ritual was unfamiliar to Western medical providers, and there is little published in the medical literature to guide Western providers who care for patients and families requesting Ruqyah. There is a need for further case studies, protocols, and institutional guidelines on how to incorporate Ruqyah at end-of-life within a Western framework of medicine. Describing existing practices and soliciting patient feedback and perspectives is key. Without knowledge of the concept of Ruqyah, the medical team missed an opportunity to support the patient and her family at a vulnerable time. The medical team was not aware that the ritual was being performed and was unable to provide appropriate reassurance from a medical standpoint. As Ruqyah is a widely available and understood request in Saudi Arabia, it was difficult to navigate a non-existent system to access these services in the United States. Additionally, if Ruqyah is not properly understood, it may be seen as a form of complementary or alternative medicine, a categorization that places the practice at the periphery, rather than at the center (Akhu-Zaheya and Alkhasawneh 2012; Atteiah et al. 2020). This marginalization minimizes its potential for efficacy. For example, although stronger randomized controlled trials are needed, Ruqyah is known to be a useful non-pharmacologic treatment for anxiety in perioperative settings (Ghiasi and Keramat 2018). Ruqyah is often the first form of self-treatment sought by Muslims toward spiritual healing and later forms a valuable last ritual at the end-of-life (AlJaffar 2023; Caksen 2023).

As the request for Ruqyah came when the patient was critically ill with no chance of cure for her underlying disease, the medical team misunderstood this request as denial of the patient’s medical status rather than the family’s attempt to balance hope with acceptance. Bedside nursing expressed a deep sense of helplessness and remorse. The family was seeking metaphysical relief, utilizing the Quran as a source of comfort toward the end-of-life and Ruqyah did not represent a lack of acceptance on the face. As the Quran states, “We send down the Quran as a healing and mercy for the believers” (The Holy Quran). The family sought to set up an end-of-life vigil, representing an Islamic practice of reminding the dying person of the beauty in the hereafter and repeating affirmations of faith. As reported by Alshammary et al. in a survey of 200 adult patients with a cancer diagnosis seeking Ruqyah at end-of-life, 154 (77%) patients identified religiosity and spirituality as their number one reason for doing so – only 4 (2%) did so for its credibility of healing and 3 (1.5%) due to dissatisfaction with conventional treatment (Alshammary et al. 2018).

This misunderstanding was a missed opportunity for openness and cultural humility, which may have revealed a known precedence for Ruqyah to achieve these goals in Islam. In fact Ruqyah is widely offered and publicized in other parts of the world both in person and virtually (Ummah Welfare Trust 2019). The use of Ruqyah to assess for magic or “possession” as a driver of the patient’s ailing health and to attain spiritual healing was likely the family’s final effort to cure the patient’s underlying terminal illness, which could have significant ramifications in the bereavement process for the family (Ummah Welfare Trust 2019). In this case, the presence of a religious and cultural interpreter may have served as a bridge between the provider team and family. Unfortunately family members are often forced into this role, whether directly or indirectly, and during the period of time of unhindered death this is a practice that should be consciously avoided. In this gray area, this space may be better held by palliative care providers and chaplaincy who can directly clarify the role of Ruqyah as end-of-life care and solicit family preferences of providing in-person recitation. Transparent and empathetic communication is key (Razzaghi and Afshar 2016). Muslim chaplaincy is the appropriate resource to navigate Islamic tradition and to ask nuanced clarifying questions to patient and family about goals of use of Ruqyah. For the medical team, this means actively communicating with the supporting teams to help facilitate Ruqyah. Therefore, for the medical provider, active and early engagement with Muslim Chaplaincy is key in understanding the role of Ruqyah and its ramifications. Along with guidance from the Palliative care team, this will contribute to the broader goal of achieving appropriate cultural competence. Although medical literature exists regarding the use of Ruqyah in the Eastern World, these are difficult to conceptualize for Western trained non-Muslim healthcare providers. Strategies such as continued education via ongoing training programs and cultural awareness initiatives should form the backbone of this endeavor. Local and community protocols and guidelines should be developed to guide medical teams when faced with requests to perform Ruqyah. For this patient, Ruqyah certainly held a role at end-of-life, and we owe it to future patients to support this ritual in hopes of healing and providing culturally sensitive care.

## Conclusion

Dying is a very challenging stage of life and it is incumbent for the medical provider team to provide support and comfort to the patient and family. An understanding of Islamic end-of-life practices is key in hospital systems that care for Muslim patients. Furthermore, providers must take active steps to involve Muslim Chaplaincy and Palliative care services at end-of-life even as it pertains to religious rituals. Discussing a patient’s specific religiosity in the context of end-of-life is key in content information gathering. It is incumbent on providers to be aware of Ruqyah guidelines when necessary. Exact details of Ruqyah are outside the scope of this paper, but information can be found in literature published from Muslim majority countries (Kulsoom 2024). There must be an acknowledgement that Muslims are not a monolith, but rather a diverse group with varying practices. A broad approach to end-of-life for this specific patient population is necessary.

## Supporting information

Thorvilson et al. supplementary material Thorvilson et al. supplementary material

## Supplementary material

The supplementary material for this article can be found at https://doi.org/10.1017/S1478951525000434.

## References

[ref1] Abdel Haleem M.A.S (2008) *The Qur’an*. Oxford University Press.

[ref2] Akhu-Zaheya LM and Alkhasawneh EM (2012) Complementary alternative medicine use among a sample of Muslim Jordanian oncology patients. *Complementary Therapies in Clinical Practice* 18(2), 121–126. doi:10.1016/j.ctcp.2011.10.003 22500850

[ref3] AlJaffar MA, Enani SS, Alhadani AH, et al. (2023) Determinants of qualify of life of cancer patients at a tertiary care medical city in Riyadh, Saudi Rabia. *Frontiers in Psychiatry* 14, 1098176. doi:10.3389/fpsyt.2023.1098176 PMC9944126 36846221

[ref4] Al-Shahri MZ and Al-Khenaizan A (2005) Palliative care for Muslim patients. *The Journal of Supportive Oncology* 3(6), 432–436. 16350430

[ref5] Alshammary SA, Duraisamy B, Al-Odeh F, et al. (2018) The satisfaction of Ruqyah on cancer patients. *International Journal of Research Studies in Medical and Health Sciences* 3(8), 1–5. ISSN: 2456-6373.

[ref6] Atteiah A, Marouf A, Alhazmi R, et al. (2020) Prevalence of complementary and alternative medicine use in brain tumor patients at King Abdulaziz University Hospital, Jeddah, Saudi Arabia. *Saudi Medical Journal* 41(6), 614–621. doi:10.15537/smj.2020.625102 32518928 PMC7502940

[ref7] Browne J, Dittborn M and Brierley J (2021) The doctrine of double effect. *Archives of Disease in Childhood Education and Practice Edition* 106(5), 304–305. doi:10.1136/archdischild-2020-320615 33452012

[ref8] Caksen H (2023) Ruqyah and its use among patients with cancer. *Journal of Child Science* 13(1), e20–e27. doi:10.1055/s-0043-1764498

[ref9] Elkadi A (1985) Health and healing in the Qur’an. *American Journal of Islamic Social Sciences* 2(2), 291–296. doi:10.35632/ajis.v2i2.2771

[ref10] Ghiasi A and Keramat A (2018) The effect of listening to Holy Quran recitation on anxiety: a systematic review. *Iranian Journal of Nursing and Midwifery Research* 23(6), 411–420. doi:10.4103/ijnmr.IJNMR_173_17 30386389 PMC6178573

[ref11] Health Ministry to certify spiritual healers *Arab News* April 20 2016 arabnews.com/islam-perspective/news/913131

[ref12] The Holy Quran 17, 81–82.

[ref13] The Holy Quran 17, 82.

[ref14] Ibn Majah Al-rabi Al-qazwin AAMIY, and Al-Khattab N (2007) *English Translation of Sunan Ibn Majah with Commentary*. Darussalam: Darussalam Publishers & Distributors.

[ref15] Ibrahim BS (2003) Spiritual medicine in the history of Islamic medicine. *Journal of the International Society for the History of Islamic Medicine* 2(4), 45–49.

[ref16] Kulsoom B (2024) Ruqyah: listening to Quranic verses, a disease treatment strategy. *International Journal of Islamic and Complementary Medicine* 5(1), 56–70. doi:10.55116/IJICM.V5I1.64

[ref17] Mayberry J (2022) Islamic Medicine: a true discipline for the 21st century or quackery? *Medico-Legal Journal* 90(1), 32–40. doi:10.1177/00258172211059919 35156431

[ref18] Muslim IA-H, and Al-Khattab N (2007) *English Translation of Sahih Muslim*. Darussalam: Darussalam Publishers & Distributors.

[ref19] Oakley J (2023) Islamic virtues and end-of-life decisions in clinical practice: a commentary on Mehrunisha Suleman, ‘The Balancing of Virtues-Muslim Perspectives on End of Life Care: empirical research analysing the perspectives of service users and providers.’ *Bioethics* 37(1), 69–71. doi:10.1111/bioe.13118 36464929 PMC10107599

[ref20] Razzaghi MR, and Afshar L (2016) A conceptual model of physician-patient relationships: a qualitative study.*Journal of Medical Ethics and History of Medicine* 9(14), 1–7. 28050244 PMC5203686

[ref21] Ruqyah: a Remedy for Illnesses, Evil Eye, Magic and Jinn from the Quran and Sunnah (2019) Ummah Welfare Trust. https://uwt.org/wp-content/uploads/2019/03/Ruqyah-Booklet (accessed 25 June 2024).

[ref22] Salim MA (2004) From the biomedical model to the Islamic alternative: a brief overview of medical practices in the contemporary Arab world. *Social Science & Medicine* 58(4), 697–702. doi:10.1016/S0277-9536(03)00221-1 14672586

[ref23] Suleman M (2023) The balancing of virtues-Muslim perspectives on palliative and end of life care: empirical research analysing the perspectives of service users and providers. *Bioethics* 37(1), 57–68. doi:10.1111/bioe.13109 36448985 PMC10099990

[ref24] Tayeb MA, Al-Zamel E, Fareed MM, et al. (2010) A “good death**”:** perspectives of Muslim patients and health care providers. *Annals of Saudi Medicine* 30(3), 215–221. doi:10.4103/0256-4947.62836 20427938 PMC2886872

