# Supplementary material for: The role of Islamic Ruqyah at end-of-life: An opportunity to provide metaphysical relief
Source: Palliat Support Care. 2025 May 23;23:e106. doi: 10.1017/S1478951525000434 (PMC13166332; doi:10.1017/S1478951525000434)
Supplement: Thorvilson et al. supplementary material [file S1478951525000434sup001.docx]

*Title*

**Reviewer 1:** The title is clear and descriptive, but consider making it more specific by highlighting the cultural and clinical integration aspects of Ruqyah.

**Response:** Thank you for this feedback, the title was edited to be more specific.

*Abstract*

**Reviewer 1:** The abstract provides a good summary but should include more details about the methodology to help readers understand the study design and analytical framework more quickly.
**Response:** Thank you for this advice, the methods section in the abstract was expanded to include more information about the methodology of the paper.

*Introduction*
**Reviewer 1:** The introduction has established the importance of the topic well. However, adding more references to previous studies on integrating Islamic practices into Western healthcare would strengthen the context and highlight the novelty of this study.

**Response:** Thank you for this feedback, additional studies were included into the introduction to provide more context of this subject matter.

*Methods*
**Reviewer 1:** The case study has been described in detail, but an additional explanation of the ethical considerations and process of engaging the chaplaincy team and spiritual care would be beneficial.

**Response:** Thank you for this advice, this was further clarified.

**Reviewer 1:** Describe in more detail how patient and family perspectives were taken and incorporated into the analyses.
**Response:** Thank you for this feedback, due to the nature of the circumstances and to be cautious and respectful of the family’s need to grieve, the details of this case were derived from a thorough chart review of the patient’s electronic medical record.

*Results and Discussion*
**Reviewer 1:** The discussion nicely highlighted the cultural and clinical challenges that the healthcare team faced. However, please include specific recommendations for practitioners on accommodating Ruqyah or similar practices. Explain how these findings contribute to the broader discussion on cultural competence in palliative care.
**Response:** Thank you for this feedback, this was further expanded in the final paragraph of the discussion.

*Conclusion*
**Reviewer 1:** The conclusions are aligned with the findings but could be strengthened by emphasizing actionable steps or recommendations for healthcare providers.

**Response:** Thank you for this advice, specific actionable steps were added to the conclusion.

*Practical Implications*
**Reviewer 1:** Although this manuscript discusses the importance of cultural sensitivity, adding specific protocols or examples from other settings where Ruqyah was successfully integrated would increase its practical relevance.

**Response:** Thank you for this advice, specific actionable steps were added to the conclusion.

*Technical Aspects*
**Reviewer 1:** Ensure consistency in referencing format and verify that all citations are in the journal's required style.

**Response:** Thank you for this feedback, this has been confirmed to be consistent.
